# Are Plant-Based Diets Detrimental to Muscular Strength? A Systematic Review and Meta-Analysis of Randomized Controlled Trials

**DOI:** 10.1186/s40798-025-00852-7

**Published:** 2025-06-02

**Authors:** Miguel López-Moreno, Eugenio Viviani Rossi, José Francisco López-Gil, Paula Marrero-Fernández, Alberto Roldán-Ruiz, Gabriele Bertotti

**Affiliations:** 1 Faculty of Health Sciences, Diet, Planetary Health and Performance, Universidad Francisco de Vitoria, Pozuelo, Spain; 2 School of Physiotherapy, Faculty of Health Sciences, Universidad Francisco de Vitoria, Madrid, Spain; 3 Faculty of Medical Sciences, UNLP, La Plata, Argentina; 4 School of Medicine, Universidad Espíritu Santo, Samborondón, Ecuador; 5 Vicerrectoría de Investigación y Postgrado, Universidad de Los Lagos, Osorno, Chile

## Abstract

**Background:**

The increasing interest in plant-based diets (PBDs) results from their beneficial impact on human health and environmental sustainability. However, the effect of PBDs on muscular strength in athletes remains unclear. This systematic review and meta-analysis evaluated the impact of PBDs on muscular strength compared to omnivorous diets in adult populations.

**Methods:**

The methodology was conducted following the Preferred Reporting Items for Systematic Reviews and Meta-Analyses (PRISMA) guidelines to ensure a comprehensive and transparent review process. Four electronic databases—MEDLINE, The Cochrane Library, Web of Science, and Scopus—were searched from their inception to September 2, 2024. Randomized controlled trials (RCTs) that evaluated the impact of PBDs on the lower body, upper body, and overall muscular strength were included. The risk of bias for the included RCTs was assessed using the Cochrane Risk of Bias 2.0 tool. Standardized mean differences (SMD) were used to estimate effect sizes, and multiple random-effects meta-analyses were conducted using an inverse variance model with Paule-Mandel adjustment.

**Results:**

Eight RCTs met the inclusion criteria, involving a total of 188 participants (46% women; mean age between 20 and 65 years). The meta-analysis indicated that there were no significant differences between PBDs and omnivorous diets in terms of upper body muscular strength (SMD, − 0.12; 95% confidence interval [CI], − 0.50 to 0.27; *n* = 146), lower body muscular strength (SMD, 0.18; 95% CI, − 0.31 to 0.67; *n* = 188), and overall muscular strength (SMD, 0.21; 95% CI, − 0.16 to 0.58; *n* = 188).

**Conclusions:**

This meta-analysis suggests that PBDs do not compromise muscular strength compared to omnivorous diets. Further investigation considering key nutrients is necessary to ascertain the long-term effects of these dietary patterns on strength outcomes.

**Supplementary Information:**

The online version contains supplementary material available at 10.1186/s40798-025-00852-7.

## Key Points

- The growing popularity of plant-based diets (PBDs) due to their health and environmental benefits has raised questions about their impact on muscular strength.
- No significant differences were found between PBDs and omnivorous diets in terms of upper body, lower body, and overall muscular strength.
- Our findings suggest that these diets are compatible with athletic performance, as they do not appear to compromise muscular strength or result in inferior improvements in strength measures.

## Background

The interest in plant-based diets (PBDs) has increased significantly in recent years due to their potential benefits for human and planetary health [1]. These are defined as dietary patterns in which foods of animal origin are totally or mostly excluded. Among PBDs, the vegan diet is distinguished by excluding all animal products. The vegetarian diet, which allows the intake of eggs and dairy products, and the flexitarian diet, which incorporates animal products on an occasional basis, represent other dietary patterns within the broader category of PBDs [2]. A survey conducted in 2021 estimated that 3.4% of Europeans follow a vegan diet, 11.1% are flexitarians and 23% limit their meat consumption [3]. In the United States, the prevalence of vegan, vegetarian, and flexitarian diets has been reported with figures rising from 4 in 2019 to 11.8% in 2022 [4].

PBDs are gaining attention in sports contexts and particularly in strength sports. For instance, vegetarian athletes tend to have a higher carbohydrate consumption than omnivores [5]. As carbohydrates are the main energy source in high-intensity exercises [6], this particularity might be related to enhancements in sports performance. In addition, PBDs are rich in antioxidants and phytochemicals, which help reduce acute oxidative stress and inflammation produced during physical exercise [7]. Nonetheless, a lack of awareness and prevalent misconceptions about the impact of plant-based diets on performance may discourage athletes from considering them as a viable option for optimizing strength development [8]. Although key nutrients such as protein and iron are typically consumed in adequate amounts, concerns remain regarding the sufficiency of this dietary pattern for supporting sports performance [9]. Similarly, plant protein differs from animal protein concerning digestibility and amino acid profile. For example, compared to animal proteins, many plant proteins contain lower levels of leucine, an essential amino acid that plays a crucial role in muscle protein synthesis [10]. From a musculoskeletal perspective, the lower leucine content and digestibility of plant proteins may influence muscle protein synthesis rates, potentially affecting muscle mass maintenance and hypertrophy [11]. Additionally, the bioavailability of non-heme iron might be reduced in PBDs, which might potentially impact oxygen-carrying capacity [12]. This situation has been speculated to be related to a higher risk of anaemia, particularly in women, thus negatively affecting athletic performance [13]. Moreover, dietary components such as omega-3 fatty acids, creatine, and vitamin B12—nutrients that are less abundant in PBDs—play a crucial role in neuromuscular function and motor unit recruitment, which could negatively impact athletic performance in individuals following PBDs [14]. Although performance outcomes have been suggested to be similar between PBDs and omnivorous diets [15, 16], there has been speculation that potential differences in performance could result from varying intakes of key nutrients in each diet. Therefore, it is recommended that individuals adhering to a PBDs ensure adequate intake of these nutrients to optimize muscle strength outcomes [17].

Previous systematic reviews have evaluated the effects of PBDs on muscular strength, reporting no differences compared to omnivorous diets [5, 15, 16]. Despite the growing interest in PBDs among athletes, no meta-analysis of randomized controlled trials (RCTs) has yet examined the impact of these diets on muscular strength in comparison with omnivorous diets. A previous meta-analysis evaluated the effects of PBDs on aerobic and strength performance [16]. However, this work included RCTs, epidemiological studies, and multi-arm studies, which were included as many times as there were possible comparisons. In this manner, failing to account for the correlation between estimated intervention effects from multiple comparisons leads to a unit-of-analysis error. Therefore, following the recommendations outlined in the Cochrane methodology, the appropriate approach to avoid this unit-of-analysis error is to combine the groups in question to create a single pair-wise comparison [18]. Hence, this systematic review and meta-analysis aimed to assess the effect of PBDs on muscular strength compared to omnivorous diets in adult population.

## Methods

### Review Design

A systematic review with meta-analysis was carried out in accordance with a pre-defined protocol. The process was conducted and documented following the Preferred Reporting Items for Systematic Reviews and Meta-Analyses (PRISMA) guidelines. Additionally, the protocol was registered in the International Prospective Register of Systematic Reviews (PROSPERO) under the registration number CRD42024576245. Initially, the effect on strength outcomes and muscle mass was intended to be evaluated. However, due to the heterogeneity in how these variables were reported in the included studies (fat-free mass, lean mass, and muscle mass), the meta-analysis focused primarily on strength outcomes.

### Search Strategy and Eligibility Criteria

The following search strategy was used in MEDLINE and adapted to the other used databases (Cochrane, Web of Science and Scopus): (“Diet, Plant-Based” [MeSH Terms] OR “plant based*” OR “vegan*” OR “vegetarian*” OR “lacto-ovo*” OR “lactoovovegetarian*” OR “lactovegetarian*” OR “ovovegetarian*” OR “macrobiotic*” OR “fruitarian*” OR “herbivor*” OR “meatless*” OR “meat free*”) AND (“Resistance Training”[MeSH Terms] OR “Muscle Strength”[MeSH Terms] OR “Weight Lifting”[MeSH Terms] OR “Skeletal Muscle Enlargement”[MeSH Terms] OR “muscle, skeletal”[MeSH Terms] OR “weight lift*” OR “strength*” OR “weight train*” OR “resistance exercis*” OR “Physical Fitness” OR “lean body mass*” OR “musculoskeletal*” OR “muscle*” OR “lean body*” OR “Body composition*” OR “fat free mass*” OR “athletic performance*” OR “sport performance*”).

The detailed search strategies for each database are presented in Table S1. In line with international standards, no language restrictions were applied. The search covered all available literature from the inception of the databases up to September 2, 2024. The inclusion criteria for study selection are outlined in Table S2.

### Study Selection and Data Extraction

Articles meeting the defined criteria underwent a two-stage screening process 1). After the removal of duplicates, two independent reviewers (GB and ARR) conducted an initial screening of titles and abstracts to assess eligibility based on the selection criteria. Studies passing this stage were then subjected to full-text screening, which was also independently carried out by the same reviewers. At this level, key study details were extracted: authorship, publication year, study location, design, sample size, follow-up duration, mean age, comparator interventions, and key outcomes (lower body strength, upper body strength, or overall muscular strength). These data are summarized in Table 1. In addition, the reference lists of all included studies were reviewed to identify any additional relevant research, but no other research matching the selection criteria was found. Discrepancies regarding study eligibility at any stage were resolved by consensus among the research team (MLM, ARR, GB).

**Table 1:** Summary characteristics of the included studies

Study	Country	Study design/Duration	Duration	Population/Health status	Plant-based intervention	Comparator intervention	Results Intervention vs. control
Haub et al. 2002 [27]	USA	Randomized Controlled Trial	12 weeks	21 healthy older men (65 ± 5 y)	Lacto-ovo-vegetarian diet (0.6 g protein/kg/day soy-based protein) + resistance training (3 d/wk)	Lacto-ovo-vegetarian beef containing diet (0.6 g protein/kg/day beef) + resistance training (3 d/wk)	No significant differences between groups in upper (seated chest press, arm pull) and lower (unilateral seated leg extension, unilateral seated leg flexion, bilateral leg press) limbs strength
Burke et al. 2003 [30]	Canada	Randomized Controlled Trial	8 weeks	42 healthy adults (18 vegetarian/24 non-vegetarian) (32.5 ± 3 y) (22 women/20 men)	Vegetarian + creatine + 20–30 min exercise (3–5 d/wk) Vegetarian + placebo + 20–30 min resistance training (3–5 d/wk)	Non-vegetarian + creatine 20–30 min exercise (3–5 d/wk) Non-vegetarian + placebo 20–30 min resistance training (3–5 d/wk)	No significant differences between groups lower (knee flexion and extension) limbs strength
Wells et al. 2003 [28]	USA	Randomized Controlled Trial	12 weeks	21 healthy older men (7 women/14 men)	Vegetarian diet (0.6 g protein/kg/day protein meat-analog) + resistance training (3 d/wk)	Beef containing diet (0.6 g protein/kg/day beef) + resistance training (3 d/wk)	The intervention group significantly increased lower limb strength (knee extension) compared to control group, while no differences were detected in the seated leg curl and double leg press. No significant differences between groups were found in upper limb strength (seated chest press and seated arm pull)
Haub et al. 2005 [29]	USA	Randomized Controlled Trial	12 weeks	21 healthy older men (65 ± 5 y)	Lacto-ovo-vegetarian diet (0.6 g protein/kg/day soy-based protein) + resistance training (3 d/wk)	Lacto-ovo-vegetarian beef containing diet (0.6 g protein/kg/day beef) + resistance training (3 d/wk)	No significant differences between groups in upper (seated chest press, arm pull) and lower (unilateral seated leg extension, unilateral seated leg flexion, bilateral leg press) limbs strength
Son Lee et al. 2017 [31]	South Korea	Pilot Randomized Controlled trial	10 days	30 female undergraduate students (20.0 ± 1.1 y)	Vegetarian Diet + aerobic, flexibility and resistance training (3 h/day)	Normal lifestyle	Back (back extension), upper limb (handgrip), and lower limb (leg extension) strength increased significantly in the intervention group compared to the control group
Durkalec-Michalski et al. 2022 [32]	Poland	Randomized Controlled Trial	4 weeks	20 healthy adults (30.7 ± 3.3 y) (12 women/8 men)	Vegan diet + high-intensity functional training (3 d/wk)	Customary Mixed Diet + high-intensity functional training (3 d/wk)	No significant differences between groups were found in upper and lower (squat, deadlift) limbs strength
Roberts et al. 2022 [26]	USA	Crossover Randomized Controlled Trial	4 weeks	11 healthy adults (26.9 ± 4.3 y) (5 women/6 men)	Whole food plant-based + running or resistance training (3–4 d/wk) Plant-based meat alternatives (PBMA) + running or resistance training (3–4 d/wk)	Omnivorous diet, favoring red meat and poultry (Animal) + running or resistance training (3–4 d/wk)	No significant differences between interventions groups (WFPB and PBMA) and control group were found in upper limb strength (push-up, pull-up, chest press and lateral pull down) and lower limb (leg press) strength
Monteyne et al. 2023 [33]	UK	Randomized Controlled Trial	10 weeks	22 healthy adults (24 ± 1 y) (11 women/11 men)	Vegan diet + resistance exercise (5 days/wk)	Omnivorous diet + resistance exercise (5 days/wk)	Upper limb strength (incline bench press) increased significantly in the intervention group compared to the control group No significant differences between groups were found in lower limb strength (deadlift, barbell back squat, and knee extensor peak isometric torque)

UK, United Kingdom; USA, United States of America; wk, weeks

### Outcomes Data Collection

The main objective of this meta-analysis was to determine the pre-post intervention changes in lower body, upper body, and overall muscular strength when comparing PBDs with omnivore diets. The overall muscular strength was defined as the average of the results obtained in lower body and upper body strength assessments, as well as physical tests involving full-body muscular strength. In studies where lower body or upper body strength was assessed using different tests, a pooled analysis of the means and standard deviations was conducted using the Psychometrica software [19]. If studies provided relevant data solely in graphical form, Web Plot Digitizer was utilized for data extraction [20]. Therefore, the reported effects result from the following equation: plant-based change (post–pre)—omnivore change (post–pre). When the 95% confidence interval (CI) was reported instead of SD, SD was calculated with the following equation: “SD = (E*√*n*)/Z*2”; being E = half the width of the CI, and Z = critical value of the standard normal distribution. Additionally, concerning the data of pre-post standard deviation (SD) difference, it was reported by “SD = √((A^2 + B^2) − (2*CF*A*B))”, being A = pre SD; B = post SD; and CF = correlation factor. In this respect, the 0.79 standardized reported correlation factor was used. Crossover studies were interpreted as follows: when washout periods were not reported, the initial baseline data provided were also used as pre-intervention values for arms after the first intervention.

### Risk of Bias Assessment

The risk of bias for the included RCTs was assessed using the Cochrane Risk of Bias 2.0 tool (RoB 2.0). This tool evaluates bias across five domains: the randomization process, deviations from intended interventions, missing outcome data, measurement of the outcome, and selection of the reported result. Based on these assessments, the studies were classified into three categories: (1) Low risk of bias: all domains were judged to have a low risk of bias; (2) Some concerns: at least one domain indicated some concerns, but no domain was rated as having a high risk of bias; and (3) High risk of bias: at least one domain was rated as having a high risk of bias, or multiple domains raised concerns.

### Publication Biases

To evaluate the impact of small-study effects and potential publication bias, the Doi plot and the Luis Furuya-Kanamori (LFK) index were employed [21]. The LFK index was utilized to assess asymmetry, with a value lower than 1 indicating no asymmetry, values between 1 and 2 suggesting minor asymmetry, and values of 2 or higher indicating significant asymmetry [21].

### Statistical Analysis

Standardized mean differences (i.e., Hedges’ *g*) were employed to compute the effect size of each intervention, comparing changes in muscular strength between intervention and control groups in individual RCTs [22]. Multiple independent random-effects meta-analyses were conducted to evaluate the impact of interventions incorporating PBDs on muscular strength, including overall, lower body, and upper body measurements. The overall effect size estimate and its 95% CI were determined using a random-effects inverse variance model with Paule-Mandel adjustment [23].

To validate the findings’ reliability, sensitivity analyses were performed by sequentially omitting one study from the overall estimates. Finally, despite the limited number of studies (fewer than 10) in the meta-analysis, which prevented conducting a meta-regression for each outcome [24], an exploratory meta-regression using mixed-effects models was conducted to test whether mean age, duration of intervention, daily protein intake (g/day), daily protein intake adjusted for body weight (g/kg/day), or type of exercise included in the analysis influenced the effect estimates. The analyses were executed using R version 4.3.0 (R Core Team) and RStudio version 2023.03.1 (Posit). The *meta* package (commands *metagen*, *forest*, and *metainf*) and *metasens* package (command *lfkindex*) were utilized for the study [25]. Statistical significance was set at a two-sided *p*-value < 0.05.

## Results

**Fig. 1:**
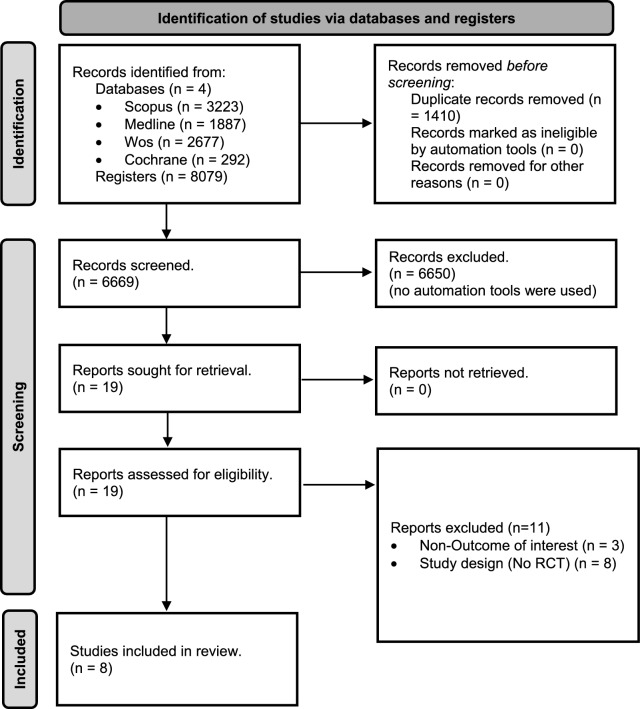
Flow diagram of study selection. WOS, Web of science; RCT, Randomized controlled trial

Of the 8079 records identified, 19 reports were assessed for eligibility and eight RCTs were finally included (Fig. 1). Reasons for exclusion at the full-text stage are presented in Table S3. Most RCTs were conducted as parallel-group trials, except for one crossover design study [26]. The studies were published between 2003 and 2023 and were carried out in the USA [26–29], Canada [30], South Korea [31], Poland [32] and the UK [33]. A total of 188 participants (46% women) were included, with sample sizes ranging from 11 to 42 participants, with an average age between 20 and 65 years old. The included participants were sedentary individuals [27–29, 31], recreational athletes [26, 30, 33] and CrossFit-trained participants [32].

The duration of the interventions ranged from 10 days to 12 weeks. Out of the eight RCTs included in the study, five employed a vegetarian diet [27–31], and three utilized a vegan diet [26, 32, 34]. In one of the RCTs, two vegan interventions based on either a whole food PBDs or plant-based meat alternatives were employed [26]. In terms of dietary recommendations, five studies employed a normocaloric diet [27–30, 32], two permitted ad libitum intake [26, 31], and one prescribed an energy surplus [33]. Across all studies, the dietary intervention was combined with a strength training programme [26–30, 33], high-intensity functional training [32] or a combined programme including aerobic training, flexibility and resistance training [31]. Finally, three of the included studies were supported by the Cattlemen’s Beef Board and the National Cattlemen’s Beef Association [27–29], and one by Marlow Foods Ltd [33].

**Fig. 2:**
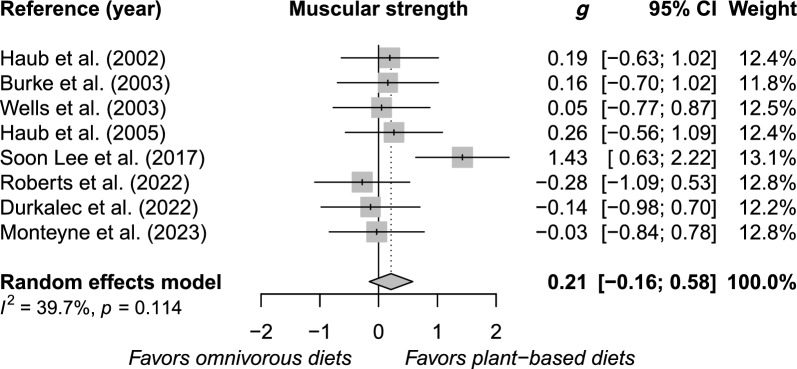
Random effects model meta-analysis for changes in muscular strength comparing plant-based diet interventions and omnivorous diets. g, Hedges’g; CI, confidence interval; I2, inconsistency index

The meta-analysis included a total of 7 RCTs for upper body muscular strength (Figure S1), 8 RCTs for lower body muscular strength (Figure S2), and 8 RCTs for overall muscular strength (Fig. 2). Results showed no differences in upper body muscular strength (SMD, − 0.12; 95% CI, − 0.50 to 0.27; *n* = 7), lower body muscular strength (SMD, 0.18; 95% CI, − 0.31 to 0.67; *n* = 8) or overall muscular strength (SMD, 0.21; 95% CI, − 0.16 to 0.58; *n* = 8) when comparing PBDs with omnivore diets.

### Sensitivity and Meta-Regression Analysis

The results of the sensitivity analyses are provided in Figure S3. Upon removal of each study from the analyses, no relevant changes were observed in the main results (regarding the direction of the estimates and the statistical significance). Additionally, meta-regression analyses did not show a moderating effect as a function of mean age (standardized beta coefficient [*B*] =  − 0.004, *p* = 0.742), duration of intervention (B =  − 0.043, *p* = 0.365), daily protein intake (*B* = − 0.009, *p* = 0.469), daily protein intake adjusted for body weight (*B* = − 0.429, *p* = 0.385), and type of exercise: aerobic and resistance training (*B* = 0.551, *p* = 0.194) and HIIT (*B* = − 0.403, *p* = 0.464) (Table S5).

### Risk of Bias in Studies

The risk of bias of the included studies was evaluated using the RoB 2.0 (Figure S4). Four RCTs were classified as exhibiting a low risk of bias [27, 29, 32, 33], two demonstrated some concerns of bias [26, 28], and two were identified as presenting a high risk of bias [30, 31].

### Publication Bias

Figure S5 shows the results of the publication bias of the included RCTs. No asymmetries were found for none of the muscular strength outcomes (LFK index, 0.66).

## Discussion

This systematic review and meta-analysis found no significant differences in upper body strength, lower strength, or overall muscular strength between PBDs and omnivorous diets. Given the growing interest in PBDs due to their potential for improving human and planetary health, our results show that these dietary patterns are compatible in the sports field, as they do not lead to inferior improvements in strength measures.

These findings are in line with those of previous systematic reviews and meta-analysis of RCTs and cross-sectional studies, which found no significant differences in strength performance following a PBDs [15, 16]. Maintaining an adequate energy intake is essential for sustaining optimal physical performance. PBDs tend to have lower energy density and promote satiety, making them ideal for weight loss goals and the prevention of chronic diseases [35, 36]. In contrast, previous studies have hypothesized that PBDs may be suboptimal for strength-sport athletes with high energy requirements [37]. In several of the included studies, to prevent any potential impact of weight fluctuations on strength parameters, participants were either permitted to self-select their energy intake or received personalised dietary plans [27–29, 32]. In this manner, energy intake did not show significant differences between the plant-based and omnivorous diets. Similarly, the impact of PBDs on strength parameters has been evaluated under conditions of ad libitum intake, which is a more accurate representation of a real-life scenario [26, 31]. Indeed, one of the included studies showed promising results derived from a vegetarian diet ad libitum, as greater fat loss was observed, with an improvement in strength outcomes being also found. In this article with outlier results, the increase in muscular strength observed in the PBD intervention was higher than that reported in the other studies included. The participants followed a health promotion program that included lifestyle modifications beyond the PBDs, such as a training programme, massage, and healthy cooking practices, which were not applied to the control group [31]. Therefore, given the characteristics of the study design, it is challenging to isolate the specific effect attributed to the plant-based intervention that could explain this outlier value. On the other hand, when specific recommendations were given to replace animal-derived foods with whole-food plant-based or plant-based meat alternatives, no differences in energy intake were observed between a vegan diet and an omnivorous diet [26].. Moreover, it highlights the importance of recognising animal food alternatives to make plant food choices compliant with energy needs, as prioritising energy-dense foods such as nuts, seeds or oils will facilitate meeting energy requirements.

Traditionally, it has been suggested that plant protein represents an inferior quality compared to animal protein due to its lower content of essential amino acids and reduced bioavailability, which could limit the anabolic potential of these amino acids [38]. However, the commonly used protein quality assessments that assign lower scores to plant proteins compared to animal proteins have been contested [39]. This would imply the need to increase the intake of plant protein sources as a mechanism to compensate for these limitations [40]. Previous studies suggest that a vegan diet is equally effective as a protein-matched omnivorous diet in supporting muscle strength and mass accrual, indicating that protein source does not influence resistance training adaptations in untrained young men with adequate protein intake [41]. It has been reported that switching from an omnivorous to a vegan diet without specific macronutrient prescriptions, despite leading to a lower dietary protein intake, does not appear to cause changes in upper and lower body strength performance [42, 43].The studies included in the meta-analysis showed a similar dietary protein intake [27–29, 32, 33] or lower [26, 30] among participants on PBDs (Table S4). Nevertheless, no significant changes were detected in strength outcomes. This could be attributed to the fact that in these studies, with prescribed dietary intake or ad libitum consumption, participants met or exceeded the dietary protein requirements of 0, 8 g/kg/day necessary to optimize its effect on muscle strength [44–46]. Additionally, when several sources of plant-based protein are consumed throughout the day without the need to combine them in a single meal, this can stimulate muscle protein synthesis and strength outcomes, similar to omnivorous diets [41, 47].

### Limitations

The results should be interpreted with caution for several reasons. Firstly, the included RCTs had small sample sizes and short-term follow-ups, which limits our capacity to comprehend the long-term impact of PBDs on muscular strength. Secondly, the studies were predominantly conducted on sedentary participants or recreational athletes, limiting the generalizability of the findings to other populations, such as elite athletes. Thirdly, half of the RCTs demonstrated concerns regarding bias or a high risk of bias, which could potentially impact the reliability and validity of the results. Fourthly, it was not possible to prove whether the findings were consistent across different subgroups, such as sex, race/ethnicity or general population/athletes.

## Research Gaps and Future Directions

The analysed evidence remains insufficient for any definitive conclusions. Further long-term studies are required to evaluate the potential impact of PBDs on nutrient intake, body composition, and physical performance. Similarly, given the sex-based disparities in muscular strength, it is imperative to evaluate the impact of these dietary patterns on strength performance, considering the specific physiological characteristics of each sex [48]. Furthermore, it is important to investigate the influence of these dietary regimens on elite athletes, who have elevated energy demands, as this could potentially represent a challenge in such instances for plant-based interventions. Finally, it is also necessary to conduct further research utilising ad libitum PBDs to delve deeper into the understanding of the effects of these dietary patterns on a regular daily basis for the general population.

## Conclusion

Despite the ongoing debate regarding whether restricting animal products may diminish exercise capacity, the results of our meta-analysis provide compelling evidence that PBDs, including vegan diets, do not adversely affect muscular strength. However, given the limited literature available comparing the physical performance of individuals following omnivorous diets with those adhering to PBDs, these findings should be interpreted with caution. Further research is required to assess the long-term impact of these dietary patterns on strength outcomes.

## Supplementary Information

Supplementary material 1.

## Data Availability

This systematic review does not include original data. Data are extracted from the literature and are publicly available. The dataset generated for the present analyses are available upon request to the corresponding author.

## Abbreviations

CI: Confidence interval
LFK: Luis Furuya-Kanamori
PBDs: Plant-based diets
PRISMA: Systematic reviews and meta-analyses
PROSPERO: International prospective register of systematic reviews
RCTs: Randomized controlled trials
RoB 2.0: Cochrane Risk of Bias 2.0 tool
SD: Standard deviation
SMD: Standardised mean difference
