# Supplementary material for: Are Plant-Based Diets Detrimental to Muscular Strength? A Systematic Review and Meta-Analysis of Randomized Controlled Trials
Source: Sports Med Open. 2025 Jun 2;11:62. doi: 10.1186/s40798-025-00852-7 (PMC12130401; doi:10.1186/s40798-025-00852-7)
Supplement: Supplementary file 1 — Supplementary material 1. [file 40798_2025_852_MOESM1_ESM.docx]

**Are Plant-Based Diets Detrimental to Muscular Strength? A Systematic Review and Meta-Analysis of Randomized Controlled Trials**

**Supplementary material**

**Table S1**. Search strategy.

**Table S2**. PICOS criteria used to define the research question.

**Table S3**. Studies excluded with reasons for exclusion after the full-text reading (n=11).

**Table S4**. Protein intake (g/kg/day) by dietary interventions.

**Table S5**. Meta-regression using mixed-effects models by mean age, duration of intervention, daily protein intake, daily protein intake per kilogram of body weight per day, and type of exercise.

**Figure S1**. Random effects model meta-analysis for changes in upper body muscular strength comparing plant-based diet interventions and omnivorous diets.

**Figure S2**. Random effects model meta-analysis for lower body muscular strength changes comparing plant-based diet interventions and omnivorous diets.

**Figure S3**. Random effects model meta-analysis for body composition-related outcomes changes comparing plant-based diet interventions and omnivorous diets.

**Figure S4.** Sensitivity analysis for muscular strength one by one the different randomized controlled trials.

**Figure S5**. Risk of bias (Rob 2.0).

**Figure S6.** Luis Furuya-Kanamori (LFK) Index and Doi Plot for muscular strength.

**Table S1**. Search strategy.

| **Medline**: September 09, 2024 (1887) |
| --- |
| ("Diet, Plant-Based" [MeSH Terms] OR "plant based*" OR "vegan*" OR "vegetarian*" OR "lacto-ovo*" OR "lactoovovegetarian*" OR "lactovegetarian*" OR "ovovegetarian*" OR "macrobiotic*" OR "fruitarian*" OR "herbivor*" OR "meatless*" OR "meat free*") AND ("Resistance Training"[MeSH Terms] OR "Muscle Strength"[MeSH Terms] OR "Weight Lifting"[MeSH Terms] OR "Skeletal Muscle Enlargement"[MeSH Terms] OR "muscle, skeletal"[MeSH Terms] OR "weight lift*" OR "strenght*" OR "weight train*" OR "resistance exercis*" OR "Physical Fitness" OR "lean body mass*" OR "musculoskeletal*" OR "muscle*" OR "lean body*" OR "Body composition*" OR "fat free mass*" OR "athletic performance*" OR "sport performance*") |
| **Cochrane**: September 02, 2024 (292) |
| ("Plant-Based Diet" OR "plant based*" OR "vegan*" OR "vegetarian*" OR "lacto-ovo*" OR "lactoovovegetarian*" OR "lactovegetarian*" OR "ovovegetarian*" OR "macrobiotic*" OR "fruitarian*" OR "herbivor*" OR "meatless*" OR "meat free*") AND ("Resistance Training" OR "Muscle Strength*" OR "Weight Lifting" OR "Skeletal Muscle Enlargement" OR "muscle, skeletal" OR "weight lift*" OR "strenght*" OR "weight train*" OR "resistance exercis*" OR "Physical Fitness" OR "musculoskeletal*" OR "muscle*" OR "lean body mass*" OR "lean body*" OR "Body Composition*" OR "fat free mass*" OR "Athletic Performance*" OR "sport performance*") in Title Abstract Keyword |
| **Scopus**: September 02, 2024 (3224) |
| ( TITLE-ABS-KEY ( “plant-based diet” OR vegan OR vegetarian OR lacto_ovo OR lactoovovegetarian OR lactovegetarian OR ovovegetarianOR macrobiotic OR fruitarian OR herbivor* OR meatless* OR meat AND free* ) AND TITLE-ABS-KEY ( "resistance training" OR "muscle strength" OR "weight lifting" OR "skeletal muscle enlargement muscle skeletal" OR "weight lift" OR strenght* OR "weight train*" OR "resistance exercis*" OR "physical fitness" OR "lean body mass*" OR musculoskeletal* OR muscle* OR "lean body*" OR "body composition*" OR "fat free mass*" OR "athletic performance*" OR "sport performance*" ) ) |
| **Web of Science**: September 02, 2024 (2677) |
| TS=("Diet, Plant-Based" [MeSH Terms] OR "plant based*" OR "vegan*" OR "vegetarian*" OR "lacto-ovo*" OR "lactoovovegetarian*" OR "lactovegetarian*" OR "ovovegetarian*" OR "macrobiotic*" OR "fruitarian*" OR "herbivor*" OR "meatless*" OR "meat free*") AND TS=("Resistance Training"[MeSH Terms] OR "Muscle Strength"[MeSH Terms] OR "Weight Lifting"[MeSH Terms] OR "Skeletal Muscle Enlargement"[MeSH Terms] OR "muscle, skeletal"[MeSH Terms] OR "weight lift*" OR "strenght*" OR "weight train*" OR "resistance exercis*" OR "Physical Fitness" OR "lean body mass*" OR "musculoskeletal*" OR "muscle*" OR "lean body*" OR "Body composition*" OR "fat free mass*" OR "athletic performance*" OR "sport performance*") |
| Total: 8079 |

| **Table S2**. PICOS criteria used to define the research question. | |
| --- | --- |
| Parameter | Inclusion Criteria |
| **Patient population (P)** | Adults (>18 years old), not pregnant. |
| **Intervention (I)** | Plant based diet (ovo vegetarian, lacto vegetarian, ovo-lacto vegetarian and diet) |
| **Comparators (C)** | Omnivorous diet, non-intervention, dietary advice or habitual diet |
| **Outcomes (O)** | Lower body strength, upper body strength or overall muscular strength |
| **Study design (S)** | Randomized controlled trials |

**Table S3**. Studies excluded with reasons for exclusion after the full-text reading (n=11).

| **ID** | **Reference** | **Reason for exclusion** |
| --- | --- | --- |
| 1 | Raben A, Kiens B, Richter EA, Rasmussen LB, Svenstrup B, Micic S, et al. Serum sex hormones and endurance performance after a lacto-ovo-vegetarian and a mixed diet. Med Sci Sports Exerc. 1992;24(11):1290–7. | Study design (no RCT) |
| 2 | Barton Jr. ML, Evans WJ, Beard JL, Campbell WW. Effects of a lacto-ovo-vegetarian diet and resistance training on body composition in older men. FASEB Journal. 1998;12(5):A965. | Study design (no RCT) |
| 3 | Campbell WW, Barton ML, Cyr-Campbell D, Davey SL, Beard JL, Parise G, et al. Effects of an omnivorous diet compared with a lacto-ovo-vegetarian diet on resistance-training-induced changes in body composition and skeletal muscle in older men. Am J Clin Nutr. 1999;70(6):1032–9 | Study design (no RCT) |
| 4 | Baguet A, Everaert I, De Naeyer H, Reyngoudt H, Stegen S, Beeckman S, et al. Effects of sprint training combined with vegetarian or mixed diet on muscle carnosine content and buffering capacity. Eur J Appl Physiol. 2011;111(10):2571–80 | Non-outcome of interest |
| 5 | Veleba J, Matoulek M, Hill M, Pelikanova T, Kahleova H. “A Vegetarian vs. Conventional Hypocaloric Diet: The Effect on Physical Fitness in Response to Aerobic Exercise in Patients with Type 2 Diabetes.” A Parallel Randomized Study. Nutrients 2016, Vol 8, Page 671. 2016;8(11):671 | Study design (no RCT) |
| 6 | Villano I, La Marra M, Messina A, Di Maio G, Moscatelli F, Chieffi S, et al. Effects of vegetarian and vegan nutrition on body composition in competitive futsal athletes. Progress in Nutrition. 2021 Feb 7;23(2). | Study design (no RCT) |
| 7 | Hevia-Larraín V, Gualano B, Longobardi I, Gil S, Fernandes AL, Costa LAR, et al. High-Protein Plant-Based Diet Versus a Protein-Matched Omnivorous Diet to Support Resistance Training Adaptations: A Comparison Between Habitual Vegans and Omnivores. Sports Medicine. 2021 Jun 1;51(6):1317–30. | Study design (no RCT) |
| 8 | Isenmann E, Eggers L, Havers T, Schalla J, Lesch A, Geisler S. Change to a Plant-Based Diet Has No Effect on Strength Performance in Trained Persons in the First 8 Weeks—A 16-Week Controlled Pilot Study. International Journal of Environmental Research and Public Health. 2023;20(3):1856. | Study design (no RCT) |
| 9 | Sathiaraj E, Afshan K, Sruthi R, Jadoni A, Murugan K, Patil S, et al. Effects of a Plant-Based High-Protein Diet on Fatigue in Breast Cancer Patients Undergoing Adjuvant Chemotherapy - a Randomized Controlled Trial. Nutr Cancer. 2023;75(3):846–56 | Non-outcome of interest |
| 10 | Cárcamo-Regla R, Zapata-Lamana R, Ochoa-Rosales C, Martorell M, Carrasco-Marín F, Molina-Recio G. Effectiveness of Resistance Training Program on Body Composition in Adults Following Vegan Diet versus Omnivorous Diet; Developed in Mobile Health Modality. Nutrients. 2024;16(15):2539. | Study design (no RCT) |
| 11 | Presti N, Rideout TC, Temple JL, Bratta B, Hostler D. Recovery after Exercise-Induced Muscle Damage in Subjects Following a Vegetarian or Mixed Diet. Nutrients. 2024;16(16). | Study design (no RCT) |

 RCT, Randomized controlled trial.

| **Study** | **Plant-based intervention**  **(g/kg/day)** | **Comparator intervention**  **(g /kg/day)** |
| --- | --- | --- |
| Haub et al 2002 [27] | 1.15 ± 0.1 | 1.03± 0.3 |
| Burke et al. 2003 [30] | 1.16 ± 0.03 | 1.99 ± 0.02 |
| Wells et al. 2003 [28] | 1.1 ± 0.2 | 1.1 ± 0.2 |
| Haub et al. 2005 [29] | 1.15 ± 0.1 | 1.03 ± 0.3 |
| Son Lee et al. 2017 [31] | - | - |
| Durkalec-Michalski et al. 2022 [32] | 1.53 ± 0.3 | 1.79 ± 0.2 |
| Roberts et al. 2022 [26] | 1.09 ± 0.2 | 1.57 ± 0.4 |
| Monteyne et al. 2023 [33] | 1.6 ± 0.5 | 1.6 ± 0.4 |

**Table S4**. Protein intake (g protein/kg body weight/day) by dietary interventions.

Values ± Standard deviation

**Table S5**. Meta-regression using mixed-effects models by mean age, duration of intervention, daily protein intake, daily protein intake per kilogram of body weight per day, and type of exercise.

| **Variables** | ***k*** | ***B*** | **SE** | **LLCI** | **ULCI** | ***p*** |
| --- | --- | --- | --- | --- | --- | --- |
| Mean age (years) | 7 | –0.004 | 0.013 | –0.030 | 0.022 | 0.742 |
| Duration (weeks) | 8 | –0.043 | 0.048 | –0.137 | 0.050 | 0.365 |
| Protein intake (g/d) | 7 | –0.009 | 0.012 | –0.033 | 0.015 | 0.469 |
| Protein intake (g/kg/day) | 7 | –0.429 | 0.494 | –1.396 | 0.539 | 0.385 |
| Type of exercise | 8 |  |  |  |  |  |
| *Resistance training* | Ref. |  |  |  |  |  |
| *Aerobic/resistance training* |  | 0.551 | 0.424 | –0.280 | 1.381 | 0.194 |
| *HIIT* |  | -0.403 | 0.551 | –1.482 | 0.676 | 0.464 |

HIIT, high-intensity interval training; *k*, number of studies; LLCI, lower limit confidence interval; Ref, reference.

**Figure S1**. Random effects model meta-analysis for changes in upper body muscular strength comparing plant-based diet interventions and omnivorous diets.

**Figure S2**. Random effects model meta-analysis for lower body muscular strength changes comparing plant-based diet interventions and omnivorous diets.


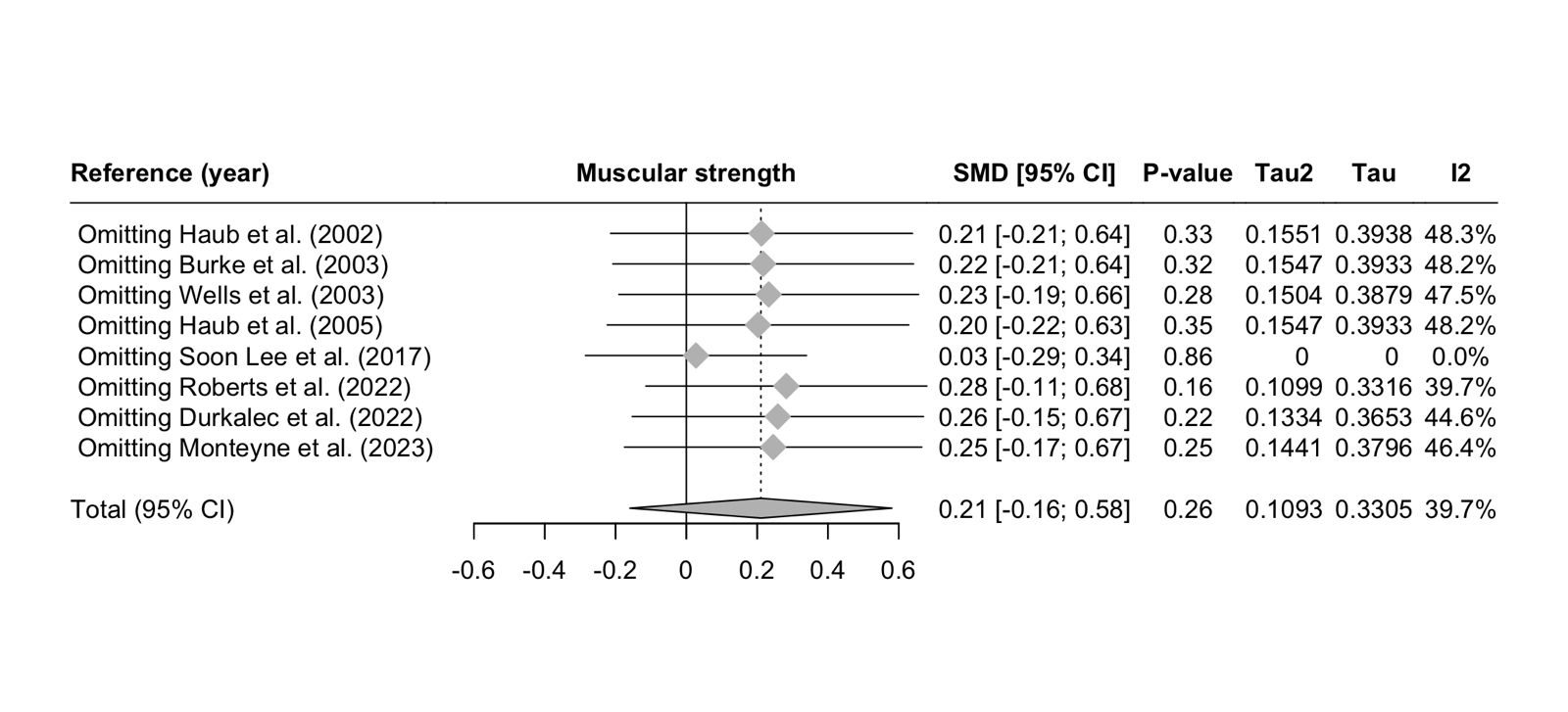
 **Figure S3.** Sensitivity analysis for muscular strength one by one the different randomized controlled trials.


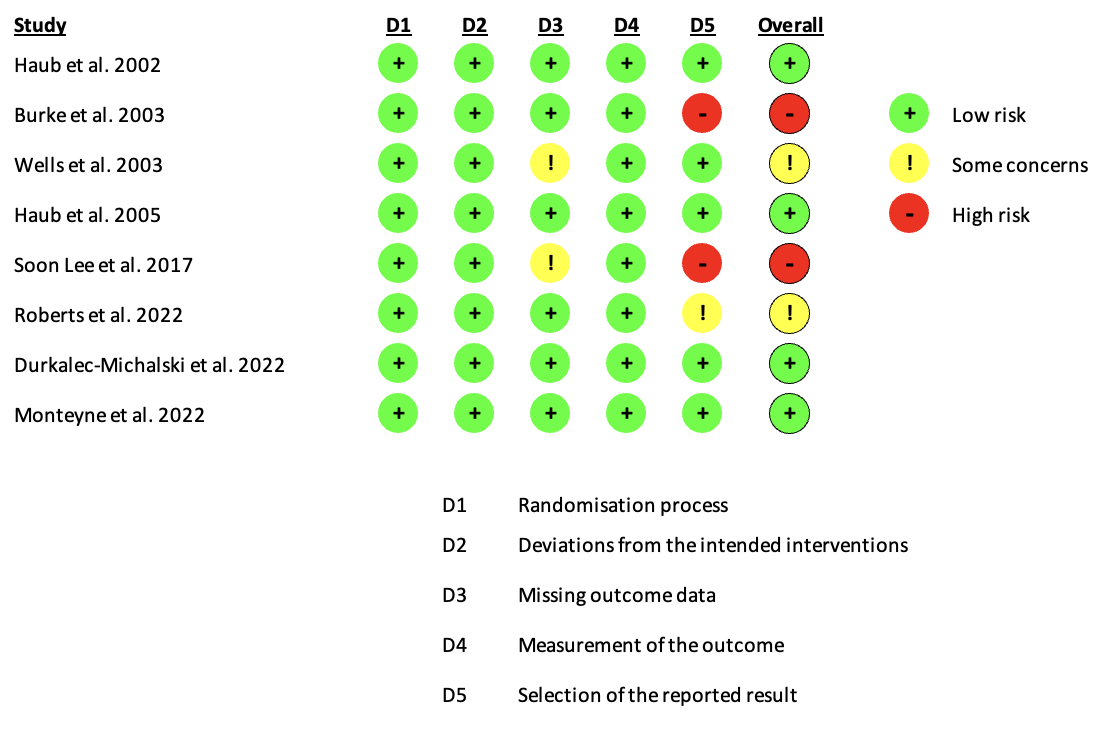
**Figure S4**. Risk of bias (RoB 2.0).


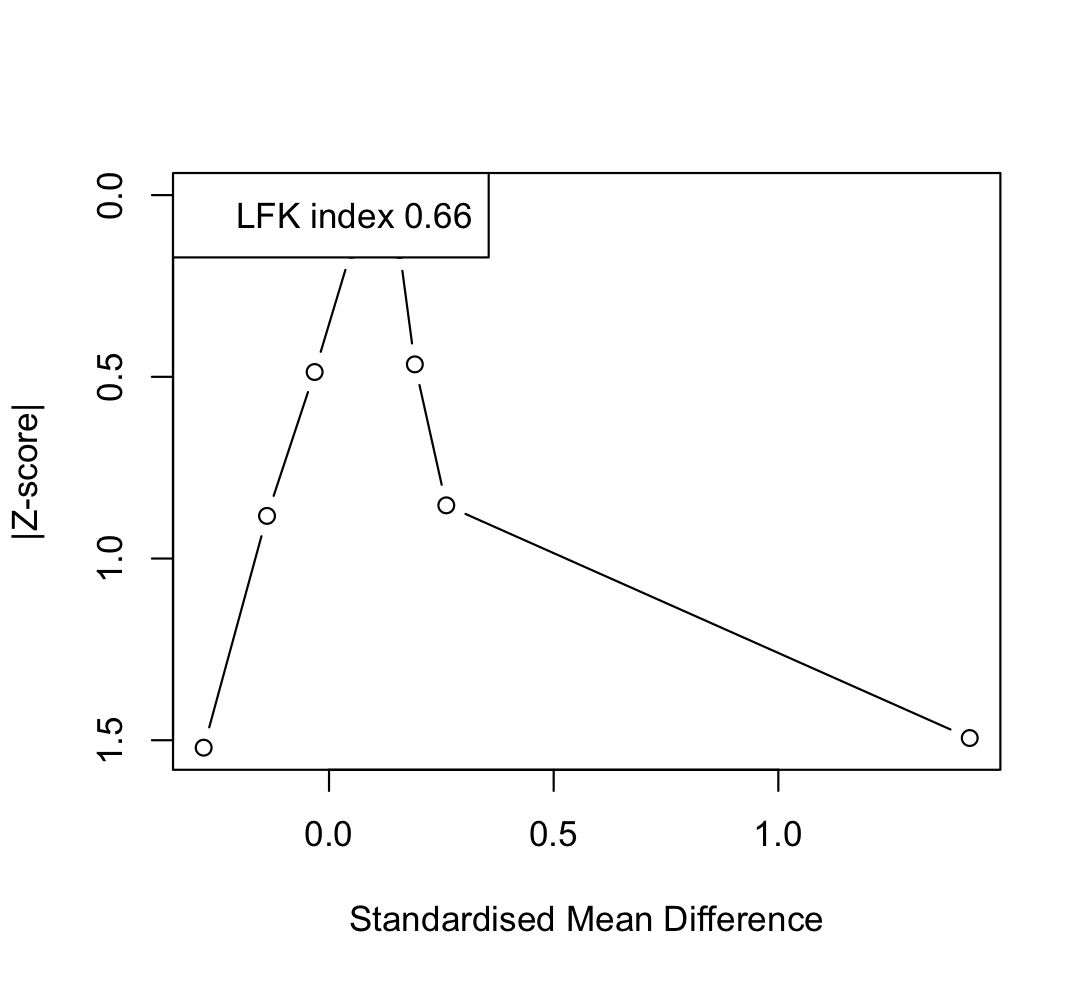
**Figure S5.** Luis Furuya-Kanamori (LFK) Index and Doi Plot for muscular strength.
